# Case Report: Totally endoscopic minimally invasive mitral valve surgery during pregnancy: a case series

**DOI:** 10.3389/fcvm.2024.1300508

**Published:** 2024-02-26

**Authors:** Zhenzhong Wang, Lishan Zhong, Haijiang Guo, Yanli Liu, Chengbin Zhou, Yingxian Ye, Fengzhen Han, Huanlei Huang

**Affiliations:** ^1^ Department of Cardiac Surgery, Guangdong Provincial People’s Hospital (Guangdong Academy of Medical Sciences), Southern Medical University, Guangzhou, China; ^2^ Department of Obstetrics, Guangdong Provincial People’s Hospital (Guangdong Academy of Medical Sciences), Southern Medical University, Guangzhou, China; ^3^ Department of Anesthesia, Guangdong Provincial People’s Hospital (Guangdong Academy of Medical Sciences), Southern Medical University, Guangzhou, China

**Keywords:** total endoscopy, cardiac valve surgery, pregnancy, cardiopulmonary bypass, minimally invasive

## Abstract

A totally endoscopic minimally invasive approach is widely used for cardiac valve surgery in normal adults. However, minimally invasive cardiac surgery during pregnancy is rarely reported. In addition to traditional median thoracotomy, totally endoscopic minimally invasive approaches can now be used for pregnant patients. We describe our experience with totally endoscopic cardiac valve surgery (TECVS) during pregnancy, which is safe for both mothers and fetuses.

## Introduction

Heart disease is the primary cause of maternal and fetal death in 1%–4% of pregnancies (1). Cardiac surgery is recommended only when medical therapy or interventional procedures fail and the mother's life is threatened (2). This is likely due to the difficulty of performing cardiac surgery during pregnancy and the complexity of multidisciplinary cooperation with important departments, such as obstetrics. We herein introduce our approach to TECVS during pregnancy.

## Description of the cases

In all patients, general anesthesia was managed. Patients were placed in the 20° counterclockwise lateral decubitus position with a mat under the right shoulder. The right upper arm was positioned horizontally to the thorax. A venous cannula was placed in the superior cava vein through the jugular vein by an anesthesiologist. The cannula for cardiopulmonary bypass (CPB) was passed through the femoral vessel. After a 2 cm–2.5 cm long skin incision was made in the right groin, the femoral vein was sutured to the femoral artery with one layer of 3-0 Prolene sutures and two layers of purse sutures before puncture at the midpoint under transesophageal echocardiography (TOE) guidance. The guidewires were removed after TOE-guided confirmation that the venous catheter had been placed in the right atrium and that the arterial catheter had been placed in the ascending aorta.

**Figure 1:**
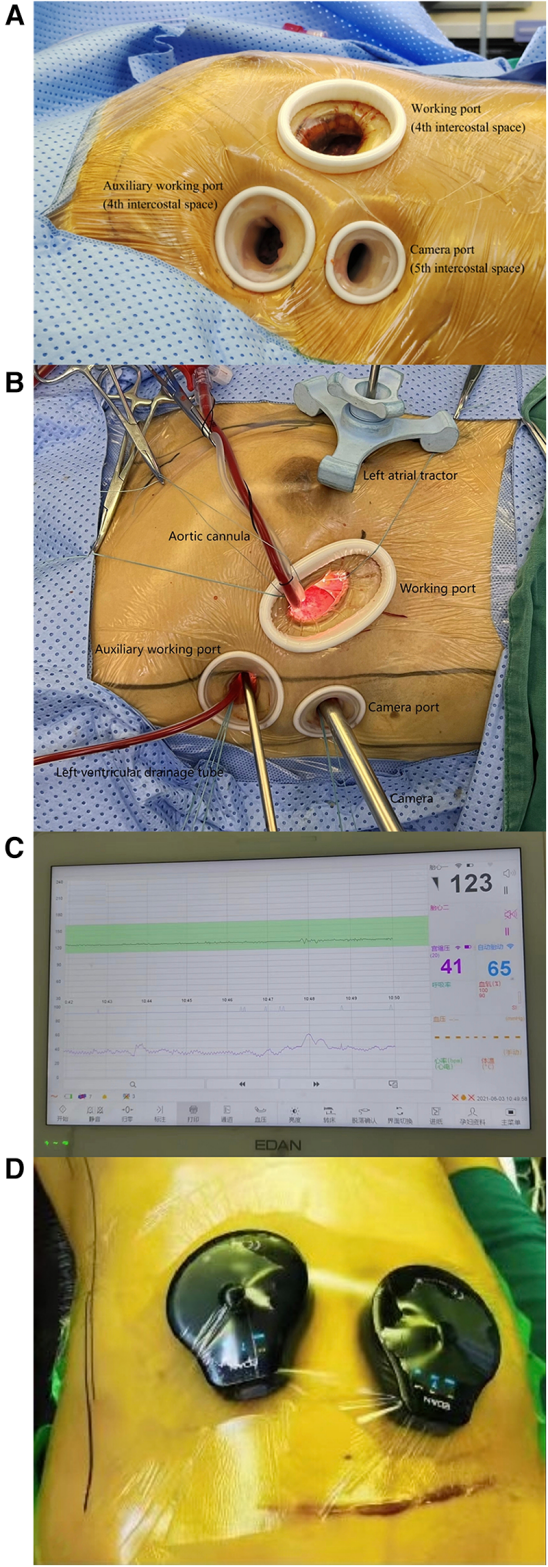
The figure shows the spatial layout of the 3-port minimally invasive cardiac surgery technique (**A,B**). Continuous fetal heart rate monitoring during surgery (**C,D**).

The 3-port minimally invasive cardiac surgery technique was mastered (Figure 1A). The system includes a main working port, an auxiliary working port and one camera port. This technique is significantly less likely to damage the mammary gland. A two-dimensional (30° oblique view) thoracoscope was inserted through the camera port. The aortic cannula was placed through the main working port, and the left heart drain was placed through the auxiliary working port (Figure 1B). Fetal heart rate was monitored throughout the procedure (Figures 1C,D). The technique allows real-time intraoperative monitoring of specific fetal heart rate changes as well as contraction pressures.

To reduce the impact of CPB, we did not start CPB until the aortic purse string suture was completed. We proposed pulsatile flow perfusion, normothermic perfusion (>35°C) and high-flow rate (>2.5 L/min/m^2^) perfusion. In addition, phenylephrine can be used to maintain a systolic pressure ≥70 mmHg to ensure placental perfusion. To minimize the duration of the procedure as much as possible, experienced surgeons and nurses must collaborate effectively during surgery. Patients were weaned from CPB as soon as possible, and the femoral vessel cannula was removed. Warfarin was used for anticoagulation, and the international normalized ratio (INR) was monitored weekly or every 2 weeks. Low-molecular-weight heparin was used for one week before delivery.

## Results

**Figure 2:**
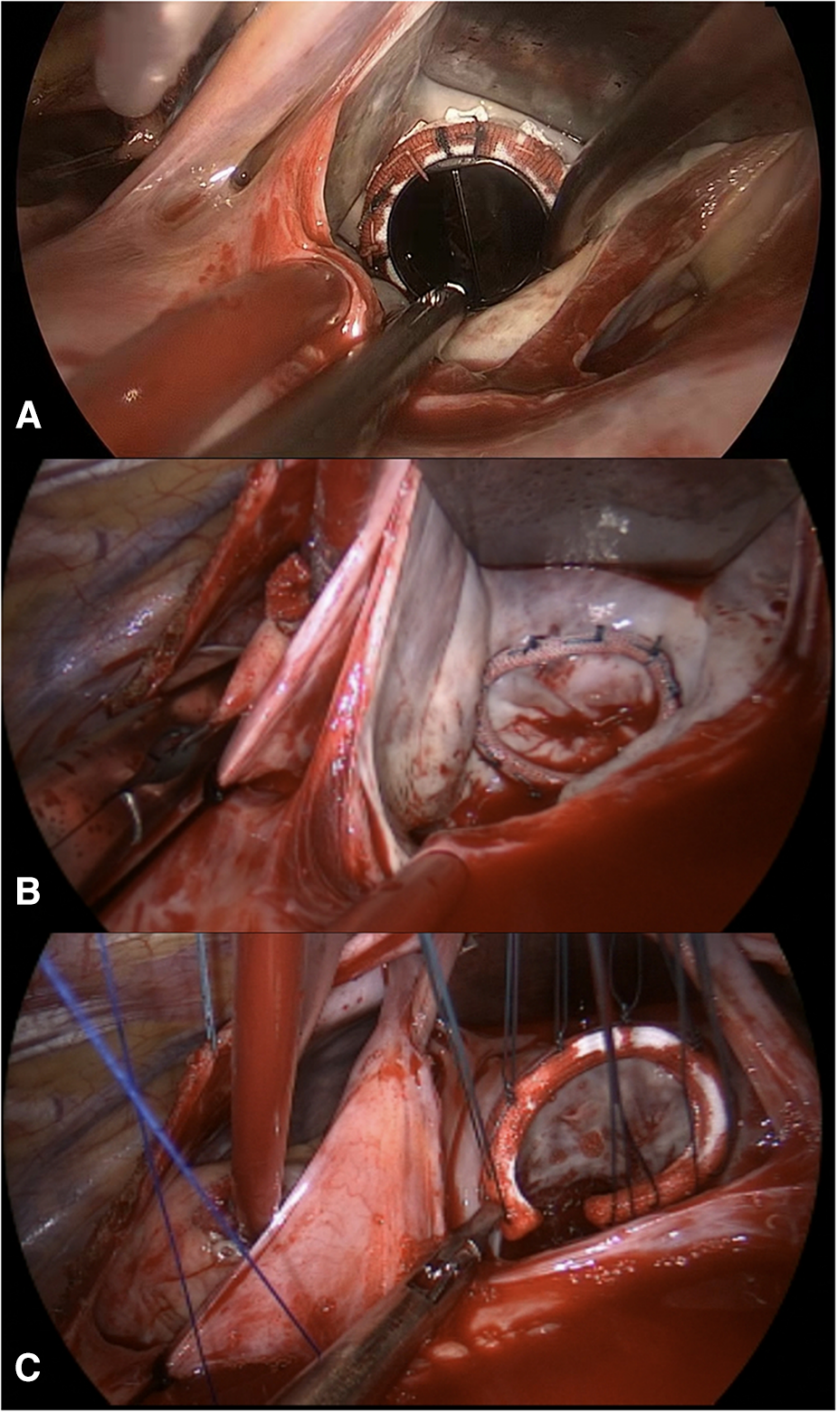
The figure shows the results of mitral valve replacement (**A**), mitral annuloplasty (**B**), and tricuspid annuloplasty (**C**).

From January 2019 to September 2022, a total 9 patients, including 4 advanced age mothers, underwent totally endoscopic cardiac valve surgery (Table 1). Eight patients were categorized as NYHA class II, and one patient was NYHA class III. Mitral valve replacement was performed in 6 (66.7%) patients. One (11.1%) patient underwent mitral valve repair, tricuspid repair and left atrial appendage closure (Figures 2A–C). The CPB time, aortic cross-clamp (ACC) time and thoracic drainage volume during the first 24 h after surgery were 89.7 ± 13.4 min, 58.3 ± 11.5 min and 260.0 ± 126.1 ml, respectively. The durations of mechanical ventilation, intensive care unit (ICU) stay and postoperative hospital stay were 3.7 ± 2.9 h, 33.3 ± 22.8 h and 5.3 ± 2.2 days, respectively. Six (66.7%) pregnant women delivered via cesarean section. Only one of the newborns was born prematurely (34^+5^ weeks). Both the 1- and 5-minute APGAR scores for all the newborns was 10. Vaginal bleeding occurred in the operating room immediately after surgery in a pregnant woman (16^+3^ weeks), and fetal ultrasound revealed no fetal heart rate. In addition, there were two cases of fetal loss after discharge. Two months after surgery, labor was induced for Patient No. 1 due to fetal nervous system abnormalities. Patient no. 2 underwent induction of labor because of personal reasons 6 days after surgery (Table 2).

**Table 1:** Preoperative details of the individual patients.

Patient no.	NYHA class	mWHO class	Age (years)	GA (weeks)	Cause of disease	Diagnosis	LVEF (%)	PAP (mmHg)
1	II	IV	28	21^+6^	IE	Severe MR, moderate MS	60	80
2	II	IV	39	18^+6^	RHVD	Severe MS, moderate MR	62	45
3	II	IV	37	18^+5^	RHVD	Moderate to severe MS, Moderate to severe MR	63	62
4	III	IV	34	32	RHVD	Severe MS, mild MR	76	60
5	II	IV	28	24	RHVD	Moderate to severe MR, moderate MS	72	57
6	II	IV	43	23	RHVD	Severe MR, mild MS	73	54
7	II	IV	36	21	RHVD	Moderate MS, moderate MR, severe TR, PFO	59	37
8	II	IV	28	16^+2^	RHVD	Extremely severe MR	65	48
9	II	II–III	33	27^+4^	RHVD	Severe MR	74	30

NYHA, New York Heart Association; GA, gestational age; IE, infective endocarditis; RHVD, rheumatoid heart valve disease; MR, mitral regurgitation; MS, mitral stenosis; TR, tricupid regurgitation; PFO, patent foramen ovale; PAP, pulmonary arterial pressure.

**Table 2:** Results of individual patients.

Patient No.	Surgical procedure	CPB (min)	ACC (min)	MV (h)	ICU (h)	PODS (d)	Outcome	
							Maternal	Fetal
1	MVR (STJ.#25)	92	64	5	39	5	Survived	Died, 35 weeks
2	MVR (STJ.#27)	75	47	4	22	8	Survived	Died, 20 weeks
3	MVP (EL.#26)	90	69	6	22	5	Survived	Alive, full term
4	MVR (Lanfei.#27)	67	43	10	22.5	3	Survived	Alive, full term
5	MVP (EL.#26)	77	45	3	29	5	Survived	Alive, full term
6	MVR (EL.#27)	96	57	0	37	4	Survived	Alive, premature
7	MVP (EL.#30)	96	57	1	19	5	Survived	Alive, full term
	TVP (EL.#28)							
	LAAO, PFOC							
8	MVP	102	63	40	94	10	Survived	Died, the day after surgery
9	MVP	112	80	2	15	3	Survived	Alive, full term

CPB, cardiopulmonary bypass; ACC, aortic cross-clamp; MV, mechanical ventilation; ICU, intensive care unit; PODS, postoperative days; PFOC, patent foramen ovale closure; MVR, mitral valve replacement; MVP, mitral valvuloplasty; TVP, tricuspid valvuloplasty; LAAO, left atrial appendage occlusion.

## Discussion

Compared with median thoracotomy, TEVAR has more advantages for normal patients, such as smaller wounds, less pain, less blood loss and shorter hospital stays (3). TECVS is a suitable option for pregnant women. The integral sternum allows better upper extremity movement and does not restrict weight lifting so that the mother can feed the baby (4). Moreover, scars were less noticeable. In addition, the efficacy of TECVS was not affected by the patient's weight, especially obese pregnant patients, and the mitral and tricuspid valves are better exposed in TECVS (5). Moreover, less postoperative pain is good for maintaining intrauterine pregnancy, which is critical for fetal survival and growth.

Pregnant patients and fetuses are significantly less tolerant of cardiac surgery than healthy adults are. The timing of surgery is crucial. In terms of fetal safety, if the operation starts too early, fetal teratogenesis (6) can easily occur, possibly leading to premature birth. The best period for surgery is between 13 and 28 weeks of gestation (2). However, it is worth noting that we successfully performed the surgery 32 weeks of gestation, and a full-term fetus was successfully delivered**.** However, one patient underwent surgery at 16 weeks of gestation, at which point the fetus died. Therefore, the best period for surgery deserves further study.

According to the modified World Health Organization (WHO) classification, all patients was class IV, which indicated that the maternal cardiac event rate was 40%–100% (2). The fetal mortality rate during CPB remains high (∼20%) (7). There are three unique pathophysiological changes in pregnancy during CPB: uterine contraction, placental hypoperfusion and fetal hypoxia (8). Most researchers agree that pulsatile flow perfusion, normothermic perfusion (>35°C) and high-flow rate (>2.5 L/min/m^2^) perfusion are beneficial^2^. Blood pressure was unstable according to our observations. We propose using phenylephrine to maintain a systolic pressure ≥70 mmHg to ensure placental perfusion. With the cooperation of perfusionists, it was feasible to start CPB just before ACC and end CPB as soon as possible. This approach was the most direct way to reduce CPB time.

The primary aim of TECVS is to cure cardiac valve disease while also protecting the fetus. The fetal mortality rate for cardiac surgery in pregnancy is approximately 30% (9). In our case series, 1 fetus (No. 8) died during surgery. We believe that this may be because both her CPB time and ACC time were above average and because she was in the early weeks of pregnancy. The fetuses of the other two patients died during the follow-up period. Patient No. 1 had fetal nervous system abnormalities. This case is the first in her and her husband's immediate family. She had been taking low-dose warfarin during her pregnancy. We believe that this patient's warfarin use may have caused fetal death, but we lack additional imaging evidence. Fetal death and fetal warfarin syndrome have also been reported with low-dose warfarin (10). The death of fetus No. 2 was chosen by the patient of her own accord, and there was no abnormality in the fetus at the time.

A multidisciplinary team is best suited for providing optimal support during the procedure. The participants included cardiac surgeons, obstetricians, anesthesiologists, perfusionists and nurse specialists. Anesthesiologists were responsible for ensuring a NRS score <3. Obstetricians immediately assessed the condition of the fetus after surgery and examined the patient in the hospital at least once per day. Atosiban acetate was injected to suppress uterine contractions when necessary. After discharge, transthoracic echocardiography was recommended for pregnant patients. All patients gave birth at our medical center under the care of a multidisciplinary team.

## Conclusions

To our knowledge, cardiac valve surgery during pregnancy poses a serious challenge. Our report suggested that TECVS can be an option for pregnant patients and that fetuses could be maintained with intrauterine pregnancy. Studies with more patients and longer follow-up periods will be performed in the future.

## Data Availability

The original contributions presented in the study are included in the article/Supplementary Material, further inquiries can be directed to the corresponding author.
